# Data on micrometeorological parameters and Energy Fluxes at an intertidal zone of a Tropical Coastal Ocean

**DOI:** 10.1016/j.dib.2018.09.108

**Published:** 2018-10-03

**Authors:** Yusri Yusup, John Stephen Kayode, Abbas F.M. Alkarkhi

**Affiliations:** a Environmental Technology, School of Industrial Technology, Universiti Sains Malaysia, USM 11800, Pulau Pinang, Malaysia; b Centre for Marine & Coastal Studies (CEMACS), Universiti Sains Malaysia, Pulau Pinang, Malaysia; c Malaysian Institute of Chemical & Bioengineering Technology, Universiti Kuala Lumpur, 78000 Melaka, Malaysia

## Abstract

Data on the micrometeorological parameters and Energy Fluxes at an intertidal zone of a Tropical Coastal Ocean was carried out on an installed eddy covariance instruments at a Muka head station in the north-western end of the Pinang Island (5°28’06’’N, 100°12’01’’E), Peninsula Malaysia. The vast source of the supply of energy and heat to the hydrologic and earth׳s energy cycles principally come from the oceans. The exchange of energies via air-sea interactions is crucial to the understanding of climate variability, energy, and water budget. The turbulent energy fluxes are primary mechanisms through which the ocean releases the heat absorbed from the solar radiations to the environment. The eddy covariance (EC) system is the direct technique of measuring the micrometeorological parameters which allow the measurement of these turbulent fluxes in the time scale of half-hourly basis at 20 Hz over a long period. The data being presented is the comparison of the two-year seasonality patterns of monsoons variability on the measured microclimate variables in the southern South China Sea coastal area.

## Specifications table

**Table:** 

Subject area	*Environmental Atmospheric Physics and Meteorology*
More specific subject area	*Environment and Climate Change*
Type of data	*Text file and figures*
How data was acquired	*Measurements of the microclimate variables at 20 Hz Half-hourly was achieved using the EC system. The data was collected for two years, i.e., from November 2015 to October 2017, which encompassed two annual cycles of the Monsoon seasons.*
Data format	*Filtered and analyzed*
Experimental factors	*The data were collected at a frequency of 20 Hz Half-hourly time series measurements.*
Experimental features	*The “Biomet” system of slow-response sensors that measured the microclimate variables was used to complement the eddy covariance (EC) system.*
Data source location	Muka head station in the north-western end of the Pinang Island (5°28’06’’N, 100°12’01’’E), Peninsula Malaysia.
Data accessibility	*The data is with this article as a supplementary excel file*
Related research article	1. Yusup Y, Alkarkhi AFM, Kayode JS, Alqaraghuli WAA. Statistical modeling the effects of microclimate variables on carbon dioxide flux at the tropical coastal ocean in the southern South China Sea. Dynamics of Atmospheres and Oceans. 2018. 84. 10–21. 〈https://doi.org/10.1016/j.dynatmoce.2018.08.002〉. 2. Yusup Y, Kayode JS, Alkarkhi AFM. A methodological approach to the air-sea energy fluxes data collection and analysis at the tropical coastal ocean, MethodsX. 2018. 5. 448–453. 〈https://doi.org/10.1016/j.mex.2018.05.003〉.

## Value of the data

• The data being presented here showed micrometeorological parameters and Energy budget at an intertidal zone of a tropical coastal ocean.
• The significance of data of this scale is in its usefulness for other researchers working on the frequency and intensity of the devastating effects of floods ravaging the region occasioned by the monsoon seasons.
• The research data is related to the connection amongst energy budget, global warming and climate change trends triggered by the monsoonal seasonal variability. The data acquisition processes and instrumentations is reproduceable in any region of the world.

## 1 Data

**Fig. 1:**
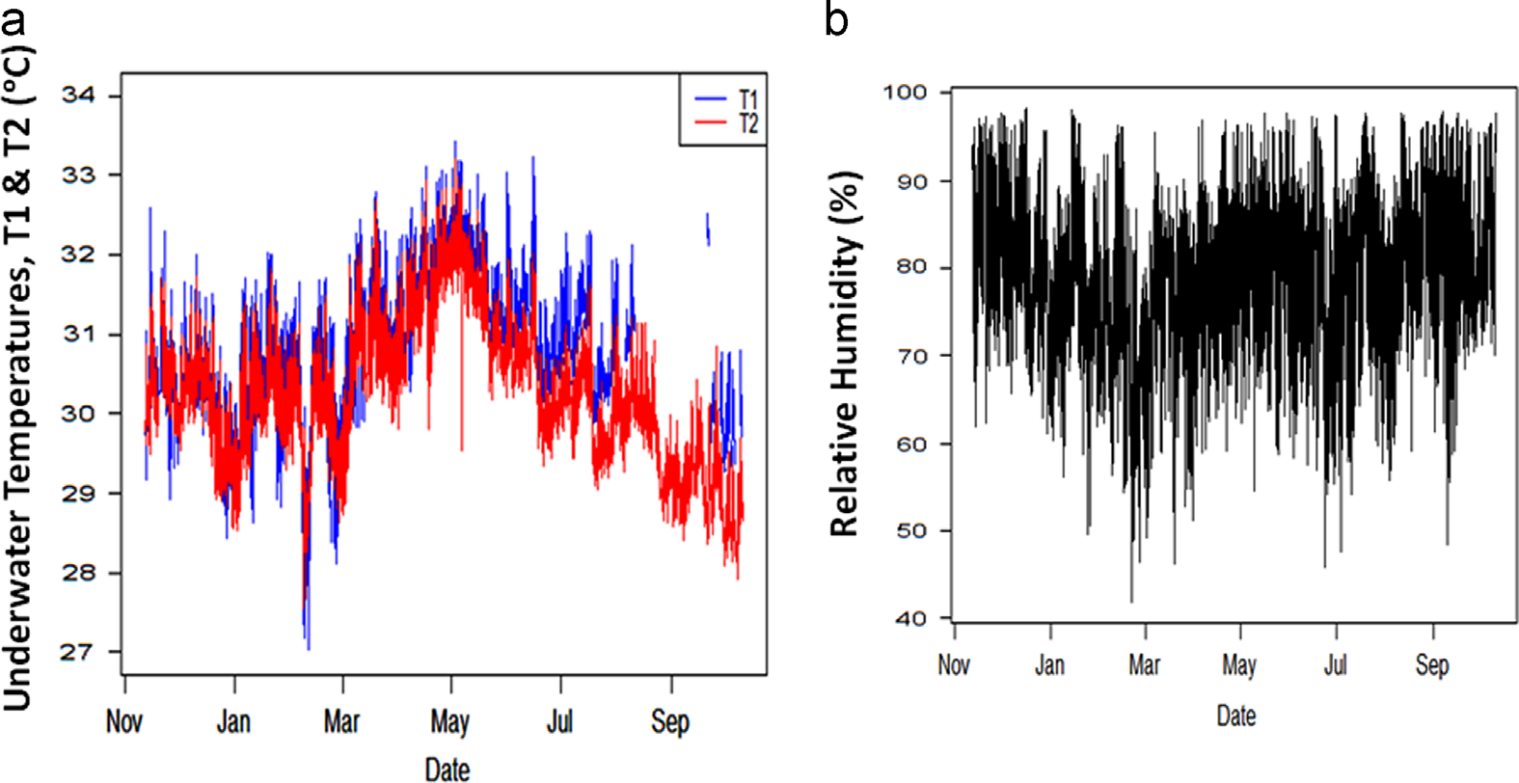
(a) Data for the Underwater temperature; *T*_1_ was measured near the surface of the ocean water, while *T*_2_ was placed deeper inside the ocean water close to the seabed. (b) Relative Humidity (RH) data in % recorded at the intertidal zone of a tropical coastal station (5.4685°N, 100.2002°E).

The data for the monsoonal variability in the tropical coast of Peninsula Malaysia on the micrometeorological parameters and Energy budget was observed based on the 2 years (i.e., from November 2015 to October 2017). The data recorded cut across two annual cycles of the Southeast Asia monsoon seasons. The data was collected to appreciate the climate variability on the overall variations of the meteorological parameters measured occasion by precipitation and temperature anomalies (Fig. 1a) brought by means of the seasonal monsoons occasioned by the continuous high vapor pressure, (Fig. 1b) [1]. A total of 33,452 data points for the period was recorded.

**Fig. 2:**
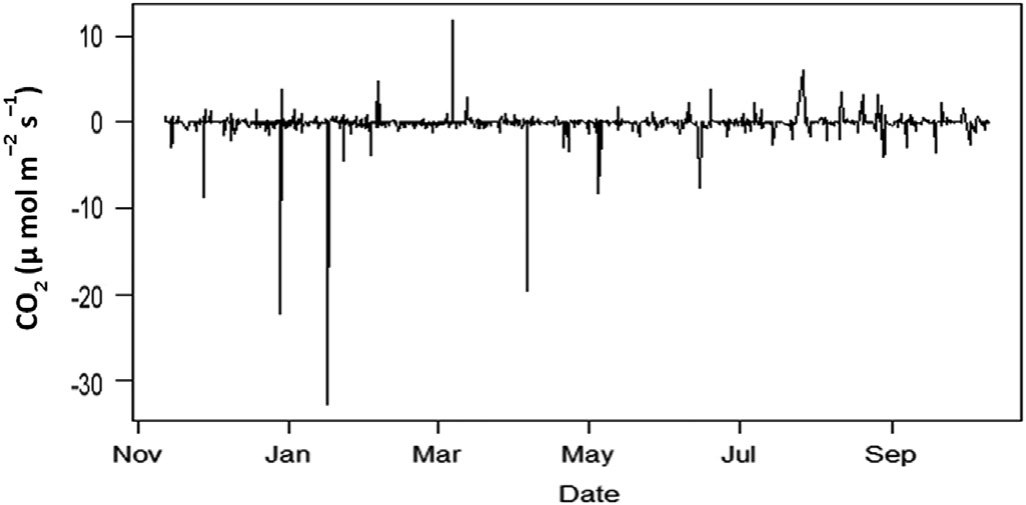
Data for the CO_2_ (µmol m^–2^ s^–1^) recorded at the intertidal zone of a tropical coastal station (5.4685°N, 100.2002°E).

The patterns of the micrometeorological data and other parameters were examined to understand the monsoon seasons on their distributions and variability (Fig. 2). Temperature variations (Fig. 1a), occasional by the number of solar radiations reaching the earth׳s surface controlled the distributions of precipitation in the southeast Asia regions [2], [3], [4]. Furthermore, the data demonstrates varied frequencies, mean, standard deviation and other statistical elements of the micrometeorological parameters between negative and positive values attributed to the extreme climate variability. Hence, there is a strong connection between the change in temperature data and the precipitation data occasioned by the monsoon meteorological conditions [4]. Statistically, it is important to understand the climate change variability due to the seasonality occasion by the monsoonal weather schemes on energy and water budget, because of the variations in Land-sea temperature caused by the high intensity of solar radiations reaching the earth surface [6]. It is this change in temperature that affected the precipitation most especially along the tropical belts.

## 2 Experimental design, materials and methods

**Fig. 3:**
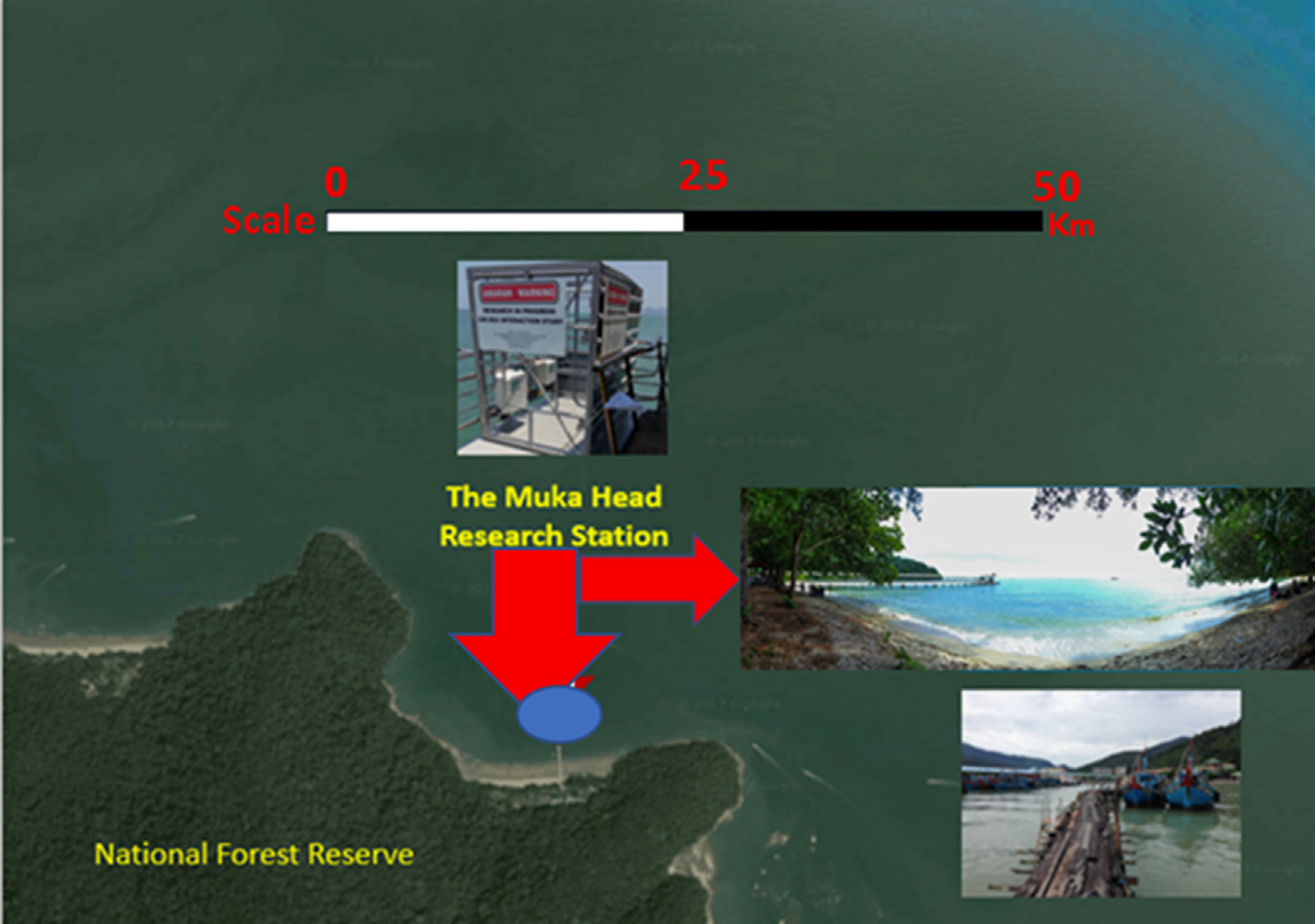
Location of the Eddy Covariance Station in Muka Head (marked by the blue circle, 5.4685°N, 100.2002°E).

The EC method used offers measurements of gas emission and ingestion that also allow measurements of energy exchanges in an area. The data collected is globally used in micrometeorological measurements in more than 3 decades. This has allowed it to grow into more advanced instrumentation and stronger practice that caused its usage across diverse disciplines and industries for environmental monitoring and inventory [5]. The EC data processing needs a lot of care and when setting up the instruments as the mathematics involved is multifaceted. The list of variables that satisfy the scientific drive for the experiment help in setting up the data acquisition instruments. The micrometeorological parameters will determine the kind of instrument to be used and the types of measurements to be made. The applicability of the data collected is a function of the site selection (Fig. 3), the instruments platform design and placement prior to measurements. The data collection and processing are streamlined according to the aims and objectives [1], [5].

Eddy Covariance (EC) is a statistical tool to compute micrometeorological parameters, turbulent fluxes, and other useful parameters to define climate variability for environmental impact purposes [6]. Although the uses of EC tools are not limited to meteorological data alone, respective drive for experimental targets will require unique settings and diverse types of variables that would be required for computation and improvements to the parameters interested to the researcher. EC depends on the direct and speedy measurements of the real gas transport using the 3-D wind speed in real time in-situ which enabled the computations of energy fluxes and micrometeorological parameters, within the atmospheric boundary layer of the southern South China Sea with respect to the effect of Southeast Asian monsoonal seasons [1], [5], [6].
